# Influence of Parental Psychological Flexibility on Pediatric COVID-19 Vaccine Hesitancy: Mediating Role of Self-Efficacy and Coping Style

**DOI:** 10.3389/fpsyg.2021.783401

**Published:** 2021-12-08

**Authors:** Yongyi Wang, Xinping Zhang

**Affiliations:** School of Medicine and Health Management, Tongji Medical College, Huazhong University of Science and Technology, Wuhan, China

**Keywords:** pediatric COVID-19 vaccine, vaccine hesitancy, self-efficacy, coping-style, psychological flexibility

## Abstract

**Background:** Pediatric COVID-19 vaccine hesitancy hinders the establishment of immune barrier in children. Psychological flexibility may be a key contributing factor to pediatric COVID-19 vaccine hesitancy, and self-efficacy and coping style play an important role in the relationship, but the underlying mechanisms remain unknown.

**Methods:** A cross-sectional study was conducted on parents from June 2021 to July 2021. A total of 382 parents were recruited for an online-investigation. Serial mediation models were used to examine whether self-efficacy and coping style mediated in the psychological flexibility-pediatric COVID-19 vaccine hesitancy linkage.

**Result:** Psychological flexibility was negatively related to pediatric COVID-19 vaccine hesitancy (*r* = −0.198, *P* < 0.001). Coping styles rather than self-efficacy played a mediating role independently (95% CI: −0.263 to −0.058). Serial mediation analyses indicated that self-efficacy and coping style co-play a serial mediating role in the association of psychological flexibility and pediatric COVID-19 vaccine hesitancy (95% CI: −0.037 to −0.001).

**Conclusion:** The present study showed that high psychological flexibility, high self-efficacy, and positive coping style were conducive to the lower pediatric COVID-19 vaccine hesitancy.

## Introduction

As of September 2021, the coronavirus (COVID-19) pandemic has caused more than 200 million confirmed cases and more than 4.5 million deaths worldwide (World Helath Organization [WHO], 2021a). Vaccine and vaccination are the most effective ways to halt the COVID-19 pandemic according to the World Health Organization (WHO) (World Helath Organization [WHO], 2021b). Currently, COVID-19 vaccination for adults and children is being promoted, but previous studies have found that vaccine hesitancy is prevalent (Bell et al., 2020; Wang et al., 2021; Yilmaz and Sahin, 2021). Vaccine hesitancy refers to the delay or refusal of vaccination despite the availability of vaccination services (Caserotti et al., 2021), which may lead to reduced vaccination rates and ultimately increased risk of infectious disease outbreaks and epidemics. Vaccine hesitancy was ranked as one of the top 10 health threats by the WHO in 2019 and deserves much attention (World Helath Organization [WHO], 2021c).

An increasing number of studies consider psychological status as one of the determinants of vaccine hesitancy, influencing vaccination willingness and behavior (Xu et al., 2021). For example, researchers found that highly anxious health-care workers tend to think that influenza vaccination is unsafe (Savas and Tanriverdi, 2010); a recent survey in Turkey showed that anxiety levels are positively related to the public’s willingness to receive the COVID-19 vaccine (Chou and Budenz, 2020). In most cases, parents are decision makers for child vaccination and their hesitancy on pediatric COVID-19 vaccine (pediatric vaccine hesitancy in short) is likely to cause children to miss or delay access to the immune barrier (Newman et al., 2018). It can be seen that the psychological status of parents may affect their willingness and decision-making for pediatric vaccines. As one of the indicators reflecting psychological status, psychological flexibility refers to the process of contacting the present moment fully as a conscious human being and persisting or changing behavior in the service of chosen values (Hayes et al., 2006). It was first proposed by American psychology professor Hayes, and became the core of Acceptance and Commitment Therapy (ACT). Psychological flexibility has now been widely used in the interpretation of health behaviors or health outcomes (Fernandes-James et al., 2019; Gentili et al., 2019; Pearlman and Thorsteinsson, 2019; Iina et al., 2021). Previous studies have shown that the psychological flexibility of adolescents with type 1 diabetes has a significant medium or strong correlation with all dimensions of quality of life (Iina et al., 2021). And the psychological flexibility of patients is strongly related to their understanding and acceptance of the correct therapeutic intervention (Pearlman and Thorsteinsson, 2019). Gentili et al. (2019) research confirmed that improving psychological flexibility as an intervention for chronic pain treatment can improve the functional status of patients, it also mentioned that psychological flexibility can also improve the self-care level of diabetic patients. Vaccination is one of the health behaviors and the relationship with psychological flexibility is well-established. A study in Hong Kong showed that the level of psychological flexibility of unvaccinated participants was significantly lower than that of participants who received influenza vaccine in the past 12 months (Cheung and Mak, 2016). On the contrary, experiential avoidance is one of the negative manifestations of psychological flexibility, that is, psychological inflexibility. Researchers have found that avoidance-based ways of regulating emotions will certainly inhibit vaccination over the past few years, such as worrying about the side effects of influenza vaccination and fear of needles (Cheung and Mak, 2016). Therefore, parental psychological flexibility may have an important impact on pediatric vaccine hesitancy.

Previous studies have found that self-efficacy and coping style are important factors influencing health behaviors, and they have also been shown to be related to psychological flexibility. College students with higher psychological flexibility had stronger self-efficacy and were more likely to make adjustments to overcome obstacles and risks they encountered (Jeffords et al., 2020). At the same time, improved self-efficacy also contributes to enhanced willingness to vaccinate. A Canadian survey of HPV vaccination intentions in different target populations, including parents of students, showed that self-efficacy was one of the determinants for parents to allow their daughters to get HPV vaccine (Gainforth et al., 2012). Christy et al. (2019) suggested that self-efficacy can indirectly increase HPV vaccination intentions (Stout et al., 2020) and is also a significant predictor of COVID-19 vaccination intentions among United States adults (Guidry et al., 2021). In terms of coping, the investigators found that coping strategy was the mediating predictor of psychological flexibility, symptoms, and quality of life in patients with psychological disorders. They believed that patients with psychological inflexibility would adopt a denial style to cope with their experiences and did not have sufficient coping skills to manage and accept them, thus exacerbating physical or emotional distress (Rueda and Valls, 2020). The psychological flexibility of the British public may partly affect the final outcome *via* coping styles (including but not limited to the promotion of more open/less avoidant responses) during the COVID-19 pandemic (Dawson and Golijani-Moghaddam, 2020). In addition, there was an association between self-efficacy and coping styles. Ma et al. (2020) stated that more negative coping styles can lead quitters to perceive themselves as unable to resist the temptation of smoking, that is, a reduction in self-efficacy. Conversely, it has also been shown that self-efficacy can influence coping styles (Geng et al., 2018; Chen et al., 2020; Liu et al., 2020).

The evidences above provide support for exploring the role of self-efficacy and coping style in the relationship between psychological flexibility and vaccine hesitancy. However, considering the possible mediating role of self-efficacy and coping style in this relationship, there is currently a lack of research focused on examining the direct or indirect effects of psychological flexibility on vaccine hesitancy.

Therefore, this study aims to investigate the relationship between parental psychological flexibility and pediatric vaccine hesitancy, and to explore the role of self-efficacy and coping styles in their association. Specifically, we propose the following hypotheses:

**Hypothesis 1.** Parental psychological flexibility significantly influences pediatric vaccine hesitancy.
**Hypothesis 2.** Self-efficacy or coping style, respectively, uniquely mediates the relationship between parental psychological flexibility and pediatric vaccine hesitancy.
**Hypothesis 3.** Self-efficacy and coping style co-play a serial mediating role in the relationship between parental psychological flexibility and pediatric vaccine hesitancy.

## Materials and Methods

### Participants and Procedures

This survey was an online questionnaire survey conducted from June 2021 to July 2021. We sent the questionnaire link to parents through the school, and parents would fill it out voluntarily. The sample size was estimated using the calculation formula of cross-sectional survey: $N\;=\;Z^{2}{{}_{1}}_{-}{{}_{\alpha }}_{/2}p(1\;-\;p)/d^{2}(\alpha \;=\;0.05,\;Z_{1}{{}_{-}}_{\alpha }{}_{/2}\;=\;1.96,\;d\;=\;0.05,\;p\;=\;30\%$ according to our pre-investigation). A total of 382 participants were included in this study, which meets the required sample size (*N* ≥ 322). In this study, 34.8% of participants were male and about 30% of participants were well educated. The participants’ mean age was 39.54 ± 5.61. A total of 86.4% of parents have get vaccinated for COVID-19, and 3.9% of parents had previously been diagnosed with COVID-19.

### Measures

#### Pediatric Vaccine Hesitancy

Pediatric vaccine hesitancy was evaluated by using the 15-item Parental Attitudes about Childhood Vaccines Survey (PACV) (Cronbach α = 0.745), which has been verified applicable and satisfactorily reliable in non-clinical settings (Opel et al., 2011a). Responses of “don’t know” for two survey items (“Have you ever delayed having your child get a shot for reasons other than illness or allergy?” and “Have you ever decided not to have your child get a shot for reasons other than illness or allergy?”) were excluded as missing data, because a “don’t know” response was felt to reflect poor recall rather than hesitant behavior. To better identify pediatric vaccine hesitancy levels, we converted this raw score to a 0–100 scale using simple linear transformation accounting for items with missing values (Opel et al., 2011b). The higher the score, the more hesitant parents are about the pediatric COVID-19 vaccine.

#### Psychological Flexibility

Acceptance and Action Questionnaire-II (AAQ-II) (Cronbach’s α = 0.884) was used to assess psychological flexibility (Bond et al., 2011). Items were reverse rated on a 7-point Likert scale ranging from 1 (always true) to 7 (never true). Higher total score (7–49) indicating higher level of psychological flexibility.

#### Self-Efficacy

The 10-item General Self-Efficacy Scale (GSES) (Zhang and Schwarzer, 1995) was applied to measure parental self-efficacy with a 4-point scale (1 “incorrect” to 4 “correct”). Higher scores indicate higher self-efficacy (Cronbach’s α = 0.897).

#### Coping Style

Coping style was assessed by the Simplified Coping Style Questionnaire (Xie, 1998) with two dimensions and each item ranges from 1 “I don’t do this” to 4 “I do it this way” (items 1–12: positive coping style; items 13–20: negative coping style) (Cronbach’s α = 0.823). The difference between the average scores of positive and negative coping styles was calculated as a variable included in the statistical analysis. The greater the difference, the more preferred the individual to positive coping style.

All participants were enrolled in the investigation using the principles of informed consent and confidentiality. The study was approved by the Ethics Committee of Tongji Medical College, Huazhong University of Science and Technology, Wuhan, Hubei, China. As the study used anonymous, pooled, and retrospective data, the ethics committee waived the need for participants to provide written informed consent. The study complies with the Declaration of Helsinki.

### Statistical Analysis

The *t*-test and one-way ANOVA were used to test the differences in pediatric vaccine hesitancy in different socio-demographic characteristics. The multiple linear regression method was used to further screen the variables that were statistically significant in the univariate analysis. Finally, age and whether have get vaccinated for COVID-19 were included as covariates into the mediation model. Pearson correlation analysis was used to test the correlation between all the main variables.

Two strategies were used to test our hypotheses and explore the pathways linking psychological flexibility to pediatric vaccine hesitancy with PROCESS 3.3. Strategy 1, parallel mediation models (Model 4) of psychological flexibility to pediatric vaccine hesitancy through self-efficacy and coping style were explored, respectively. Strategy 2, serial multiple mediator models (Model 6) were conducted to examine paths between mediators. All indirect effects were subjected to follow-up bootstrap analyses with 5000 bootstrap samples and 95% bias-corrected confidence intervals, which indicate significance when they do not include zero. Non-standardized coefficients and 95% CIs with standard errors (SE) were reported for the model. All statistical analyses were performed using IBM SPSS24.0 software.

## Results

### Bivariate Correlations Among Primary Variables

Table 1 provided the correlations among the variables studied. Pediatric vaccine hesitancy score was positively associated with psychological flexibility (*r* = 0.198, *P* < 0.001) and negatively associated with self-efficacy and coping style (*r* = −0.122, *P* < 0.05; *r* = −0.243, *P* < 0.001).

**TABLE 1:** Correlation analysis between primary variables (*n* = 382).

	Pediatric vaccine hesitancy	Psychological flexibility	Self-efficacy	Coping style
Pediatric vaccine hesitancy	1			
Psychological flexibility	−0.198***	1		
Self-efficacy	−0.122*	0.153**	1	
Coping style	−0.243***	0.379***	0.301***	1

*P < 0.05, **P < 0.01, ***P < 0.001.

### Parallel Mediation Model

**FIGURE 1:**
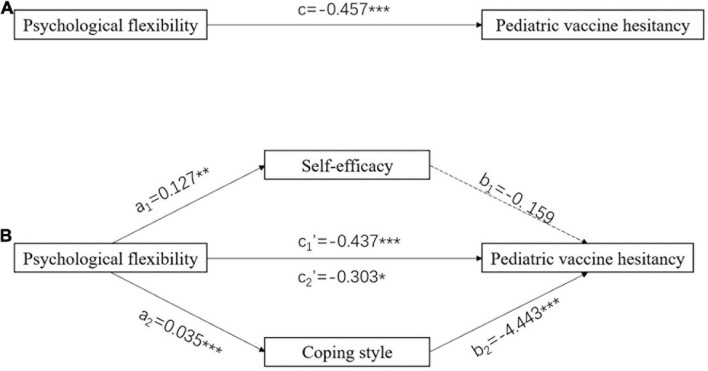
Parallel mediation model. **(A)** Total effect model. **(B)** Models with self-efficacy and coping style as mediators, respectively. Numbers associated with a, b, c, and c′ are unstandardized regression coefficients. *P < 0.05, **P < 0.01, ***P < 0.001.

The results showed that the total effect of psychological flexibility on pediatric vaccine hesitancy was significant (*c* = −0.457, *P* < 0.001) (Figure 1A). When self-efficacy and coping style was modeled as a mediator, respectively, the path coefficients revealed that coping style had a significant mediating effect (a_2_ × b_2_ = −0.154, 95% CI: −0.263, −0.058) but self-efficacy did not (Figure 1B). More details about the parallel mediation model were stated in Table 2.

**TABLE 2:** Total, direct, and indirect effect of psychological flexibility on vaccine hesitancy through self-efficacy and coping style.

Path	Estimate	SE	*t*	*P*	95% CI	
					Lower	Upper
Total effect (c)	–0.457	0.114	–3.998	0.0001***	–0.682	–0.232
**X → self-efficacy → Y**						
Direct effect (c_1_′)	–0.437	0.116	–3.779	0.0002***	–0.665	–0.210
Indirect effect (a_1_ × b_1_)	–0.020	0.022	−	−	–0.073	0.013
Indirect effect (a_1_)	0.127	0.043	2.986	0.003**	0.043	0.210
Indirect effect (b_1_)	–0.159	0.139	–1.148	0.252	–0.431	0.113
**X → coping style → Y**						
Direct effect (c_2_′)	–0.303	0.122	–2.487	0.013*	–0.543	–0.064
Indirect effect (a_2_ × b_2_)	–0.154	0.050	−	−	–0.263	–0.058
Indirect effect (a_2_)	0.035	0.004	7.950	< 0.001***	0.026	0.043
Indirect effect (b_2_)	–4.443	1.332	–3.336	0.0009***	–7.062	–1.824

X, psychological flexibility; Y, pediatric vaccine hesitancy; *P < 0.05, **P < 0.01, ***P < 0.001.

### Serial Mediation Model

**FIGURE 2:**
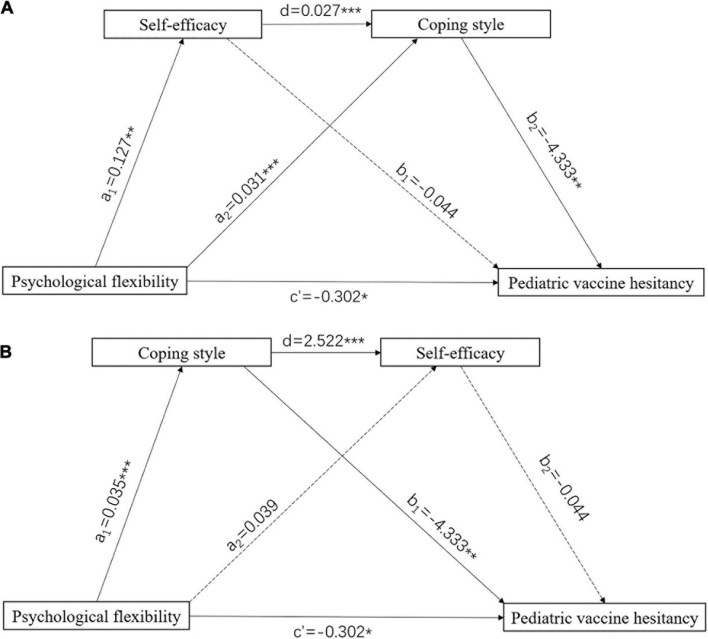
Serial mediation model. **(A)** Psychological flexibility → self-efficacy → coping style → pediatric vaccine hesitancy. **(B)** Psychological flexibility → coping style → self-efficacy → pediatric vaccine hesitancy. Numbers associated with a, b, c, and c′ are unstandardized regression coefficients. *P < 0.05, **P < 0.01, ***P < 0.001.

Figure 2 presents the coefficients and significance of each path, and Table 3 shows the results for the indirect effect. The indirect effect path (psychological flexibility → self-efficacy → coping style → pediatric vaccine hesitancy) was significant of the serial mediation model and the alternative indirect effect path (psychological flexibility → coping style → self-efficacy → pediatric vaccine hesitancy) was not significant. Self-efficacy and coping style co-play a serial mediating role in the relationship between psychological flexibility and pediatric vaccine hesitancy score, indicating that the higher psychological flexibility would enhance self-efficacy and subsequently promoted propensity for positive coping style, thereby reducing pediatric vaccine hesitancy.

**TABLE 3:** Bootstrap results of the serial mediation models.

Path	Effect	SE	*t*	*P*	95% CI	
					Lower	Upper
Total effect (c)	–0.457	0.114	–3.998	< 0.001***	–0.682	–0.232
Direct effect	–0.302	0.122	–2.467	0.014*	–0.542	–0.061
Total indirect effect	–0.156	0.052	−	−	–0.265	–0.063
**Model a**						
Indirect effect (X → self-efficacy → Y)	–0.006	0.020	−	−	–0.050	0.032
Indirect effect (X → coping style → Y)	–0.136	0.049	−	−	–0.236	–0.046
Indirect effect (X → self-efficacy → coping style → Y)	–0.015	0.009	−	−	–0.037	–0.001
**Model b**						
Indirect effect (X → coping style → Y)	–0.150	0.054	−	−	–0.263	–0.053
Indirect effect (X → self-efficacy → Y)	–0.002	0.011	−	−	–0.027	0.017
Indirect effect (X → coping style → self-efficacy → Y)	–0.004	0.012	−	−	–0.029	0.021

X, psychological flexibility; Y, pediatric vaccine hesitancy; *P < 0.05, ***P < 0.001.

## Discussion

This study explored the role of self-efficacy and coping style in the relationship between psychological flexibility and pediatric vaccine hesitancy. Mediation analysis revealed that coping style mediated the relationship of psychological flexibility and pediatric vaccine hesitancy and found a significant chain between self-efficacy and coping style.

Our findings showed that higher psychological flexibility was associated with lower pediatric vaccine hesitancy. This result was similar to a study on influenza vaccine in Hong Kong (Cheung and Mak, 2016). Previous studies have confirmed that there were many influencing factors for vaccine hesitancy, and common factors included fear emotions (such as pain) (Guidry et al., 2021), fear of vaccine side effects and vaccine efficacy (Yilmaz and Sahin, 2021) in addition to socio-demographic characteristics (Brandstetter et al., 2021). Mechanisms between psychological flexibility and vaccine hesitancy have been less studied. Theoretically, individuals with low level of psychological flexibility would choose the path with the least resistance to minimize their discomfort (Cheung and Mak, 2016), while individuals with high levels of psychological flexibility would not allow their negative thoughts or feelings to lead to avoidance or maladjustment, thereby correcting experiential avoidance and promoting positive behavioral changes (Moitra and Gaudiano, 2016). Cheung’s research showed that patients with chronic respiratory diseases with low level of psychological flexibility were more likely to refuse influenza vaccine, and at the same time reflected a high concern about vaccine side effects and infection after vaccination (Cheung and Mak, 2016). Also, improving psychological flexibility with ACT can help improve medication adherence in patients with psychosis and AIDS (Moitra and Gaudiano, 2016). A recent cross-sectional study on the development of depression and PTSD after early life trauma highlighted the importance of psychological flexibility as “it seems to adapt to change” (Gentili et al., 2019). Therefore, it can be concluded that low psychological flexibility lead to rejection and avoidance (Ramaci et al., 2019), and high psychological flexibility help overcome obstacles and risks (Jeffords et al., 2020), which in turn affect the acceptance of pediatric COVID-19 vaccine.

The results of mediation analysis showed that psychological flexibility can affect pediatric vaccine hesitancy *via* coping style. Psychological flexibility is considered as the ability to adapt to situational demands (Coyne et al., 2020), that is, individuals with high psychological flexibility will actively adjust to adapt to the environment. Such positive adjustment can be considered an embodiment of positive coping style that contribute to the self-care of cancer survivors (Geng et al., 2018), and also help children with malignant tumors treat the disease from a positive perspective (Liu et al., 2020). Yu et al. (2020) stated that women with higher levels of positive coping in the third trimester of pregnancy are less likely to suffer from postpartum depression. Yu et al. (2020) stated that women with higher levels of positive coping in the third trimester of pregnancy are less likely to suffer from postpartum depression, and the most common behaviors of these women are “paying attention to the good side of things” and so on. So, parents with more positive coping may pay more attention to the beneficial side of the pediatric COVID-19 vaccine and be more willing to make their children vaccinated.

In addition, the present study demonstrated that the serial mediation effect of self-efficacy and coping style in the relationship between psychological flexibility and pediatric vaccine hesitancy is statistically significant. Self-efficacy, as the core concept of social cognitive theory proposed by Bandura, demonstrates an individual’s ability to resist various problems and stresses in life events (Liu et al., 2020). Coping styles are personality strategies used in coping with living conditions (Geng et al., 2018), which refers to the habitual or preferred solutions or means adopted by individuals, has the function of self-protection (Liu et al., 2020), and has both positive and negative sides (Liu et al., 2020).

Consistent with a previous research (Jeffords et al., 2020), we also found that improved psychological flexibility contributed to elevated self-efficacy. This may be because psychological flexibility has something in common with self-efficacy, both involving the perceived ability to perform related activities in situations that interfere with personal experiences such as pain or distress, and emphasizing the importance of being able to persist in behavior despite experiencing negative thoughts (Jeffords et al., 2020; Schele et al., 2021). Hongyue et al. (2021) have shown that psychological flexibility played a moderating role between self-efficacy of regulatory emotional and anxiety, that is, atrial fibrillation patients with high psychological flexibility can make appropriate dynamic adjustment according to the changing situation to alleviate emotional stress, sequentially reducing the formation of negative emotions.

Simultaneously, it has been pointed out that self-efficacy can influence coping styles (Liu et al., 2020), and high levels of self-efficacy were beneficial for patients undergoing radiotherapy for head and neck cancer to adopt a positive coping style (Donghua et al., 2019). In general, the stronger the self-efficacy, the stronger the individual’s confidence in their own problem-solving ability, and the easier it is to choose a positive coping style, that is, to overcome difficulties and worries and receive the COVID-19 vaccine. Conversely, people with low self-efficacy lack confidence and will regard potential difficulties as more serious than they actually are, as well as pay too much attention to shortcomings, and finally choose negative coping style such as avoidance (Liu et al., 2020). Notably, our study did not find a significant link between self-efficacy and vaccine hesitation. Previous studies have revealed a positive (Gainforth et al., 2012) or negative correlation (Myhre et al., 2020) between self-efficacy and vaccine hesitancy. This means that the relationship between the two has not been determined.

In terms of socio-demographic characteristics, parental age and whether or not they had got COVID-19 vaccination were factors that influenced pediatric COVID-19 vaccine hesitancy. Age was negatively associated with pediatric COVID-19 vaccine hesitancy. This may be because older people have a stronger perception of disease risk (Freimuth et al., 2017). For parents who have not yet received COVID-19 vaccine, they may refuse adults and pediatric COVID-19 vaccine due to concerns about vaccine safety, complacency, and inconvenience of obtaining the vaccine (Schmid et al., 2017).

There were some limitations in our study. Firstly, the study was cross-sectional and did not establish causality. Secondly, more influencing factors associated with pediatric vaccine hesitancy should be identified in the future. Thirdly, in the actual situation, parents filled out the questionnaire while taking care of their children, it may distract the parents’ attention. Finally, we used the AAQ-II to measure parental psychological flexibility, but actually measured experiential avoidance. Other scales such as the Multidimensional Psychological Flexibility Inventory (MPFI) may be more accurate than the AAQ-II in measuring psychological flexibility (Rolffs et al., 2018), so improvements in this regard could make in future studies.

## Conclusion

This study found that psychological flexibility is a key factor influencing pediatric COVID-19 vaccine hesitancy, and both self-efficacy and coping style act as important mediators according to the serial mediation analysis. In order to achieve herd immunity as soon as possible to protect children, it is necessary to pay attention to the psychological status of parents and take relevant measures, such as ACT, cultivating and encouraging positive coping style, and psychological counseling to enhance self-efficacy.

## Data Availability Statement

The raw data supporting the conclusions of this article will be made available by the authors, without undue reservation.

## Ethics Statement

The studies involving human participants were reviewed and approved by the ethics committee of Tongji Medical College, Huazhong University of Science and Technology, Wuhan, Hubei, China. As the study used anonymous, pooled, and retrospective data, the ethics committee waived the need for participants to provide written informed consent. The study complies with the Declaration of Helsinki. Written informed consent for participation was not required for this study in accordance with the national legislation and the institutional requirements.

## Author Contributions

YW: conceptualization, methodology, formal analysis, data curation, manuscript preparation, and writing – original draft. XZ: conceptualization, validation, data collection, manuscript revision, writing – review and editing, and supervision. Both authors contributed to the article and approved the submitted version.

## Conflict of Interest

The authors declare that the research was conducted in the absence of any commercial or financial relationships that could be construed as a potential conflict of interest.

## Publisher’s Note

All claims expressed in this article are solely those of the authors and do not necessarily represent those of their affiliated organizations, or those of the publisher, the editors and the reviewers. Any product that may be evaluated in this article, or claim that may be made by its manufacturer, is not guaranteed or endorsed by the publisher.
